# Angiotensinogen-M235T as a risk factor for myocardial infarction in Asian populations: a genetic association study and a bioinformatics approach

**DOI:** 10.3325/cmj.2016.57.351

**Published:** 2016-08

**Authors:** Fariba Raygan, Mohammad Karimian, Atefeh Rezaeian, Bahareh Bahmani, Mohaddeseh Behjati

**Affiliations:** 1 Department of Cardiology, School of Medicine, Kashan University of Medical Sciences, Kashan, Iran; 2 Gametogenesis Research Center, Kashan University of Medical Sciences, Kashan, Iran; 3 Department of Molecular and Cell Biology, Faculty of Basic Sciences, University of Mazandaran, Babolsar, Iran; 4 Anatomical Sciences Research Center, Kashan University of Medical Sciences, Kashan, Iran; 5 Cardiovascular research center, Cardiovascular Research Institute, Isfahan University of Medical Sciences, Isfahan, Iran

## Abstract

**Aim:**

To investigate if there is an association between M235T polymorphism of angiotensinogen gene and myocardial infarction (MI) risk and perform a meta-analysis and an *in silico* approach.

**Methods:**

This case-control study included 340 participants (155 MI patients and 185 controls) examined at Kashan University of Medical Sciences (Kashan, Iran) between 2013 and 2015. Meta-analysis included 25 studies with 6334 MI patients and 6711 controls. Bioinformatics tools were applied to evaluate the impact of M235T polymorphism on angiotensinogen function and structure.

**Results:**

Genetic association study revealed a significant association between TT genotype (odds ratio [OR] 2.08, 95% confidence interval [CI] 1.08-4.00, *P* = 0.029) and T allele (OR 1.45, 95% CI 1.06-1.99, *P* = 0.021) and MI risk. Meta-analysis also revealed a significant association between M235T polymorphism and MI risk in allelic (OR 1.55, 95% CI 1.10-2.18, *P* = 0.012) and recessive (OR 1.69, 95% CI 1.13-2.53, *P* = 0.010) models within Asian population. *In silico*-analysis revealed that M235T fundamentally changed the function of angiotensinogen (score 32; expected accuracy 66%).

**Conclusions:**

Our study suggests that M235T polymorphism might be a helpful biomarker for screening of susceptible individuals for MI in Asian population.

Myocardial infarction (MI) is a major cause of mortality, especially in industrial countries (1). Its predisposing factors are hyperlipidemia (2), smoking (3), diabetes (4), obesity (5), and hypertension (6), but its development may also be affected by genetic factors (7,8). The key pathways involved in the MI development and progression are kallikrein-kinin and carbon metabolism pathways (9,10). Furthermore, defects in renin-angiotensin-aldosterone system (RAAS) system may result in the initiation and progression of vascular diseases. Angiotensinogen (AGT), one of the initial components in this system, interacts with renin to produce angiotensin I, the pro-hormone of angiotensin II. Genetic variations in *AGT* gene modify the plasma concentration of AGT and may be implicated in the pathogenesis of hypertension, coronary heart disease, and myocardial infarction (11,12). There is a common single nucleotide polymorphism (SNP) at location 803 (803 *t* > C; SNP ID: rs699) on exon 2 of the *AGT* gene. This transition results in substitution of methionine to threonine at codon 268, which is traditionally known as M235T. In recent years, several researchers have investigated the association between *AGT*-M235T polymorphism and MI, with inconsistent results (13-17). The aim of this study was to investigate the association between AGT-M235T polymorphism and MI risk using a case-control study and a meta-analysis followed by a bioinformatics analysis.

## Methods

### Participants

This case-control study included 340 participants: 155 patients with acute MI and 185 healthy controls. We screened about 250 patients with acute MI admitted to the Coronary Care Unit of the Shahid Beheshti Hospital (Kashan, Iran) between 2013 and 2015. The mean age ± standard deviation of patients was 62.37 ± 3.21 years (range, 56-69 years). Patients with the history of coronary artery (n = 23), vascular (n = 12), renal (n = 7), liver (n = 3), thyroid (n = 4), diabetes (n = 34), and any other familial and genetic diseases were excluded from the study. MI was confirmed by patient history, ECG changes, increased creatine kinase (CK)-MB activity, and high levels of serum troponin (T or I). The controls were selected from people who referred to the same hospital for a routine check-up. The control group included 185 healthy participants without symptoms of coronary diseases and any other familial and genetic diseases. Control participants were free from MI as shown by medical history, electrocardiography, and clinical examination. The mean age ± standard deviation of controls was 61.68 ± 4.28 years (Table 1). 2 mL of blood was drawn from each participant and preserved in complete blood count tubes at -20°C until analysis. All participants gave a written informed consent. The study was approved by the Medical Research Ethics Committee of the Kashan University of Medical Sciences.

**Table 1:** Participants’ demographic and biochemical characteristics

Variables	Case (n = 155)	Control (n = 185)	*P*
Age (years), mean ± standard deviation	62.37 ± 3.21	61.68 ± 4.28	0.098
Sex (M/F)	102/53	127/58	0.642
Smoking (Y/N)	69/86	76/109	0.582
Body mass index (kg/m^2^), mean ± standard deviation	24.65 ± 2.44	24.41 ± 2.34	0.357
High density lipoprotein (mg/dL), mean ± standard deviation	42.40 ± 4.54	41.78 ± 4.29	0.196
Low-density lipoprotein (mg/dL), mean ± standard deviation	112.62 ± 18.91	115.30 ± 14.38	0.139
Triglycerides (mg/dL), mean ± standard deviation	133.13 ± 23.98	130.02 ± 25.03	0.246
Total cholesterol (mg/dL), mean ± standard deviation	138.48 ± 24.86	141.05 ± 23.31	0.328

### DNA extraction and SNP genotyping

We analyzed just one gene polymorphism (*AGT*-M235T) by polymerase chain reaction restriction fragment length polymorphism (PCR-RFLP) method. For this purpose, total genomic DNA was isolated from blood samples by DNG-plus Kit (Cinnagen Co., Tehran, Iran). The primer sets used for amplification of *AGT* fragment were 5′-CCGTTTGTGCAGGGCCTGGCTCTCT-3′ and 5′-CAGGGTGCTGTCCACACTGGACCCC-3′. PCR was carried out in a total volume of 25 µL, including 2.5 µL 10X PCR buffer, 0.5 U of Taq DNA polymerase, 1.5 µM MgCl_2_, 0.5 µL dNTPs, 0.35 µM of each primer, and 50 ng of template DNA (all PCR reagents were purchased from Fermentas, Sankt Leon-Rot, Germany). PCR conditions were 94°C for 10 min, followed by 35 repetitive cycles of 94°C for 30 s, 59°C for 45 s, and 72°C for 60 s, with a final extension at 72°C for 10 min in a peqSTAR thermal cycler (PeqLab, Erlangen, Germany). PCR products were then digested by *Psy*I restriction enzyme (Fermentas) at 37°C for 16 h, electrophoresed on 8% polyacrylamide gel, and visualized by silver nitrate (AgNO_3_) staining as described by Green and Sambrook (18). Two fragments of 24-bp and 141-bp were observed for 235TT genotype. A single band of 165-bp was characterized as 235MM genotype. Three fragments of 24-bp, 141-bp, and 165-bp were observed for 235MT genotype. Finally, the accuracy of PCR-RFLP procedure was confirmed by DNA sequencing (Cinnagen Co.).

### Meta-analysis

Meta-analysis was conducted in accordance with the PRISMA checklist (Supplementary Table 1)(web extra material 1) and included relevant studies investigating the association between *AGT*-M235T polymorphism and MI. PubMed, ScienceDirect, and Google Scholar were searched using the following keywords: “angiotensinogen,” “AGT,” “M235T,” “polymorphism,” and “myocardial infarction.” Experimental studies were included if they applied the following criteria: (i) investigation of the *AGT-*M235T polymorphism and MI (ii) in a case-control study; (iii) on human beings; (iv) with accessible data to calculate the odds ratios (OR) and 95% confidence intervals (CI) (Table 2). The association between *AGT*-M235T polymorphism and MI was further stratified by ethnicity.

**Table 2:** Characteristics of the studies included in the meta-analysis

Country (ethnicity)	Genotype frequencies						Allele frequencies				*P*HWE*	Reference
	case			control			case		control			
	MM	MT	TT	MM	MT	TT	M	T	M	T		
France	229	301	100	258	372	111	759	501	888	594	0.219	Tiret et al 1995 (15)
Japan	6	31	66	10	41	52	43	163	61	145	0.647	Kamitani et al 1995 (13)
China	4	22	124	4	54	279	30	270	62	612	0.453	Ko et al 1997 (16)
Finland	48	66	37	53	64	34	162	140	170	132	0.088	Pastinen et al 1998 (17)
China	4	13	40	13	31	32	21	93	57	95	0.258	Chen et al 1998 (14)
UAE	14	18	8	16	26	19	46	34	58	64	0.256	Frossard et al 1998 (28)
Germany	319	582	157	385	585	222	1220	896	1355	1029	0.993	Gardemann et al 1999 (29)
Germany	38	54	30	28	53	11	130	114	109	75	0.064	Winkelmann et al 1999 (30)
Spain	69	99	52	64	96	40	237	203	224	176	0.713	Batalla et al 2000 (31)
Russia	63	85	50	43	75	34	211	185	161	143	0.905	Fomicheva et al 2000 (32)
Italy	63	124	60	54	76	27	250	244	184	130	0.976	Olivieri et al 2001 (33)
China	3	12	33	11	30	45	18	78	52	120	0.108	Xie et al 2001 (34)
Spain	59	121	32	34	97	49	239	185	165	195	0.252	Fernández-Arcás et al 2001 (35)
Turkey	32	48	22	39	59	16	112	92	137	91	0.340	Ermis et al 2002 (36)
USA	4	29	67	2	31	67	37	163	35	165	0.462	Hooper et al 2002 (37)
China	2	7	32	18	47	51	11	71	83	149	0.203	Zhu et al 2002 (38)
USA	71	98	39	215	349	153	240	176	779	655	0.608	Bis et al 2003 (39)
India	24	80	91	29	127	144	128	262	185	415	0.897	Ranjith et al 2004 (40)
England	212	252	83	197	226	82	676	418	620	390	0.208	Tobin et al 2004 (41)
China	2	10	35	13	26	31	14	80	52	88	0.087	Ren et al 2005 (42)
Brazil	46	52	12	43	51	10	144	76	137	71	0.356	Araujo et al 2005 (43)
Austria	-	-	-	-	-	-	1537	1203	832	634	-	Renner et al 2005 (44)
Poland	30	46	24	22	44	29	106	94	88	102	0.503	Konopka et al 2011 (45)
Tunisia	29	53	41	53	61	30	111	135	167	121	0.117	Mehri et al 2011 (46)
Iran	42	79	34	71	85	29	163	147	227	143	0.672	This study

*PHWE – *P* value for Hardy-Weinberg equilibrium.

### In silico analysis

The entire genomic sequence of human *AGT* gene was obtained from Ensembl (Accession No. ENSG00000135744) and analyzed using GeneRunner software (Version 5.0.43 Beta, Hastings Software, Hudson, NY, USA). The coding sequence of *AGT* gene was determined and translated to amino acid sequence by Expasy web server (*web.expasy.org/translate*). The physicochemical properties of 235M normal and 235T mutant forms were obtained from ProtParam server (*http://web.expasy.org/cgi-bin/protparam/protparam*). The impact of M235T substitution on the protein function was evaluated by SNAP (*https://rostlab.org/services/snap/*) (19), PredictProtein (*www.predictprotein.org/getqueries*) (20), and PolyPhen-2 (*http://genetics.bwh.harvard.edu/pph2/*) (21). The effects of the substitution on the secondary structure of protein were assessed by Chou-Fasman method (*http://fasta.bioch.virginia.edu/fasta_www2/fasta_www.cgi?rm=misc1&pgm=cho*). The three-dimensional structure of the AGT protein was obtained from SNPeffect 4.0 server (*http://snpeffect.switchlab.org/*) (22) and visualized using Accelrys DS Visualizer 1.7 program (*http://accelrys.com/products/discovery-studio/visualisation.php*). The Kyte-Doolittle hydropathy pattern (23) was plotted using Accelrys DS Visualizer 1.7 program.

### Statistical analysis

Hardy-Weinberg equilibrium (HWE) was calculated using χ^2^ test. The association between the MI risk and *AGT*-M235T polymorphism was estimated using odds ratios (ORs) and their 95% confidence interval (CIs), which were calculated by binary logistic regression. Odds ratios were also adjusted for age, sex, body mass index, smoking status, and biochemical features. A two-tailed *P*-value lower than 0.05 was considered statistically significant. Statistical analyses were performed using SPSS software version 19.0 (SSPS Inc., IBM Corp Armonk, NY, USA).

In the meta-analysis, we first estimated the pooled OR and 95% CI for the following five genetic models: T vs M (Allelic), TT vs MM (co-dominant), TM vs MM (co-dominant), MT+TT vs MM (dominant), and TT vs MT+MM (recessive). Values of each study were combined by random effects model (24) or Mantel-Haenszel fixed effects (25). When the heterogeneity was significant (*P*_heterogeneity_>0.1), the random effect model was used. Otherwise, the fixed effect model was used. For sensitivity analysis, each study at a time was excluded to detect the magnitude of the effect on the overall summary estimate. Begg’s funnel plot and Egger’s test were employed to evaluate the publication bias (26,27). The Open Meta Analyst (Tufts University, Medford, MA, USA; *http://www.cebm.brown.edu/openmeta/*) and Comprehensive Meta Analysis (Biostat, Inc., Englewood, NJ, USA; *https://www.meta-analysis.com/*) software were used for all calculations in the meta-analysis.

## Results

### Distribution of AGT-M235T

The distribution of *AGT*-M235T genotypes was in Hardy-Weinberg equilibrium in the MI (χ^2^ = 0.076, *P* = 0.783) and control (χ^2^ = 0.179, *P* = 0.672) group. The genotypes and allele frequencies for the M235T in the MI and control group are shown in Table 3. The frequency of MM, MT, and TT genotypes in the MI group was 27.10%, 50.97%, and 21.93%, respectively, while these ratios in the control group were 38.38%, 45.94%, and 15.68%, respectively. The frequency of M and T alleles in the MI group was 52.58% and 47.42%, respectively, while these ratios in the control group were 61.35% and 38.65%, respectively. Genotype analysis revealed a significant association between TT genotype and MI (OR 2.08, 95% CI 1.08-4.00, *P* = 0.029). Furthermore, T allele carriers (MT+TT) were at a high risk for MI development (OR 1.70, 95% CI 1.05-2.75, *P* = 0.030). Also, we observed a significant association between T allele and MI risk (OR 1.45, 95% CI 1.06-1.99, *P* = 0.021).

**Table 3:** Genotype and allele frequencies of M235T in cases and controls

Genotype/allele	No (%)		Unadjusted odds ratio (95% confidence interval)	*P*	Adjusted odds ratio* (95% confidence interval)	*P*
**controls (n = 185)**	**cases** **(n = 155)**
MM	71 (38.38)	42 (27.10)	-	-	-	-
MT	85 (45.94)	79 (50.97)	1.57 (0.96-2.56)	0.070	1.59 (0.96-2.63)	0.073
TT	29 (15.68)	34 (21.93)	1.98 (1.06-3.70)	**0.032^†^**	2.08 (1.08-4.00)	**0.029**
MT+TT	114 (61.62)	113 (72.90)	1.68 (1.06-2.66)	**0.029**	1.70 (1.05-2.75)	**0.030**
M	227 (61.35)	163 (52.58)	-	-	-	-
T	143 (38.65)	147 (47.42)	1.43 (1.05-1.94)	**0.022**	1.45 (1.06-1.99)	**0.021**

*Adjusted OR – adjusted in multivariate logistic regression models including age, sex, body mass index, smoking status, and biochemical values.

†Significant differences between the case and control groups are in bold.

### Meta-analysis

**Figure 1:**
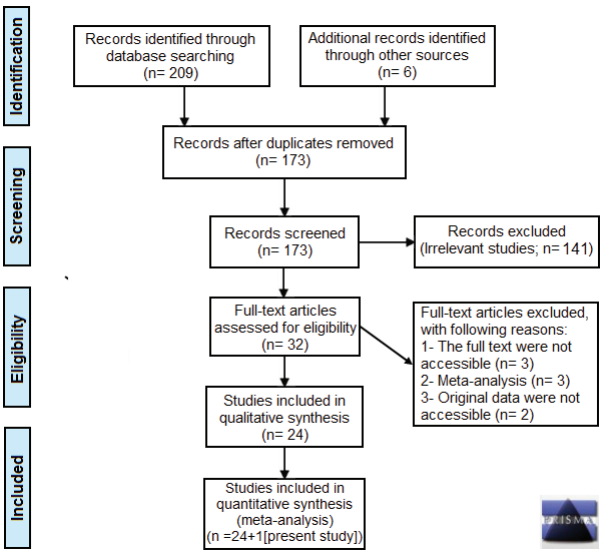
Flowchart of the study selection process.

215 articles were identified from electronic databases and the citations in potentially relevant articles. After reading abstracts and initial assessment of the articles, 42 and 141 articles were found to be duplicates and irrelevant studies, respectively. From 32 potentially relevant reports, 24 studies from 17 countries were included in the meta-analysis (13-17,28-46). 3 reports were excluded because the full-texts were not available (47-49). 3 other reports were excluded because they were meta-analyses (50-52). 2 studies did not have accessible original data (53,54). Finally, the data from our case-control study was added to the meta-analysis. Therefore, 25 studies were included in the meta-analysis, including 6711 healthy controls and 6334 patients with MI. Genotypes in all control groups were in Hardy-Weinberg equilibrium. Of the 25 studies, 9 studies involved Asian populations, 12 Caucasian, and 4 other ethnicities (Figure 1).

**Figure 2:**
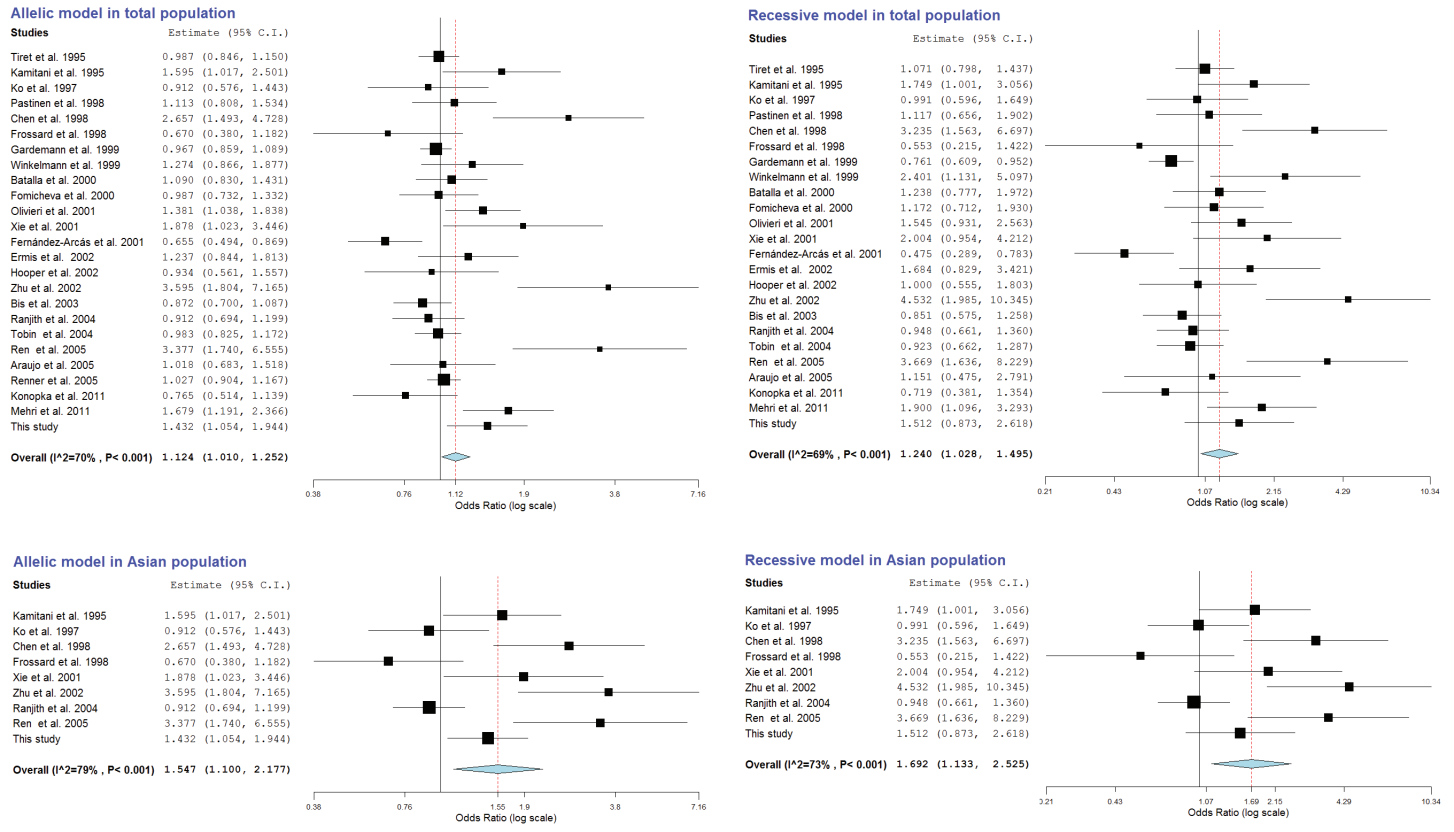
Forest plot for the association of M235T polymorphism of angiotensinogen gene (*AGT*-M235T) with myocardial infarction (MI).

The meta-analysis of 25 studies showed a significant association between M235T polymorphism and MI risk in allelic (OR 1.12, 95% CI 1.01-1.25, *P* = 0.033) and recessive (OR 1.24, 95% CI 1.03- 1.50, *P* = 0.025) models (Figure 2). In Asian population *AGT*-M235T was also associated with the MI risk in allelic (OR 1.55, 95% CI 1.10-2.18, *P* = 0.012) and recessive (OR 1.69, 95% CI 1.13-2.53, *P* = 0.010) genetic models (Figure 2). Even, after the correction of the *P-*values for multiple testing by the Benjamini-Hochberg false discovery rate method (55), *AGT*-M235T polymorphism was still significantly associated with the MI risk in Asian population (Supplementary Table 2)(web extra material 2). However, we observed no association between *AGT*-M235T and MI risk in any of the 5 genetic models in Caucasian populations (Table 4).

**Table 4:** Association results in the meta-analysis*

Group	T vs M		TT vs MM		MT vs MM		MT+TT vs MM		TT vs MM+MT	
	OR (95% CI)	*P*	OR (95% CI)	*P*	OR (95% CI)	*P*	OR (95% CI)	*P*	OR (95% CI)	*P*
Total	1.12 (1.01-1.25)	0.033	1.17 (0.94-1.46)	0.162	1.03 (0.94-1.13)	0.540	1.06 (0.93-1.22)	0.355	1.24 (1.03-1.50)	0.025
Asian	1.55 (1.10-2.18)	0.012	1.72 (0.95-3.14)	0.076	1.15 (0.86-1.55)	0.352	1.41 (0.88-2.25)	0.152	1.69 (1.13-2.53)	0.010
Caucasian	1.01 (0.92-1.10)	0.864	1.01 (0.81-1.28)	0.892	1.02 (0.92-1.14)	0.664	1.00 (0.90-1.10)	0.941	1.04 (0.83-1.29)	0.761

*OR – odds ratio; CI – confidence interval.

**Figure 3:**
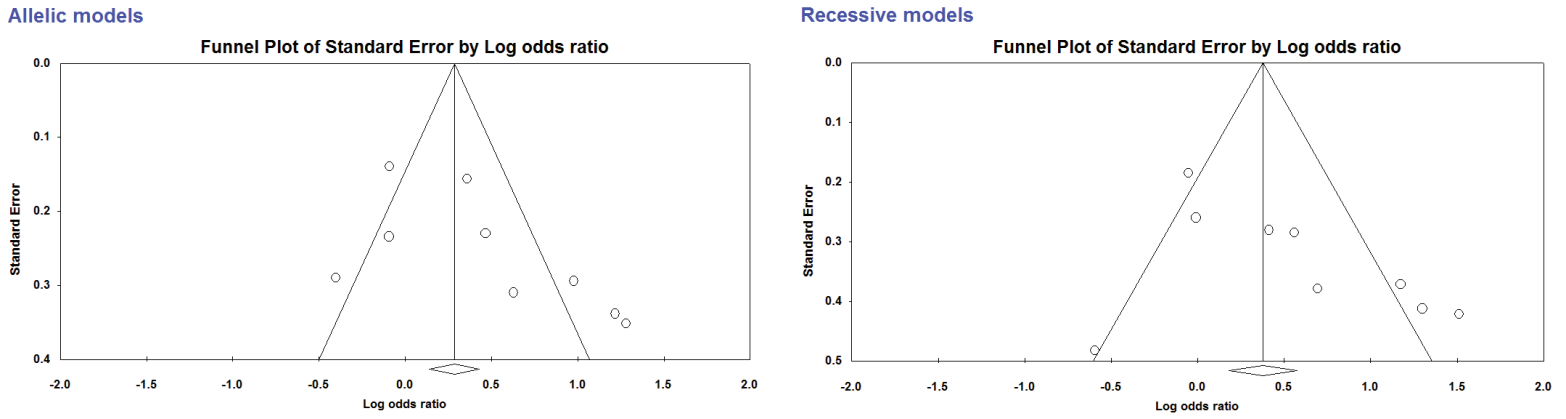
Funnel plot for association of M235T polymorphism of angiotensinogen gene (*AGT*-M235T) with myocardial infarction (MI) in Asian population.

The heterogeneity test showed a true heterogeneity between studies in the T vs M (*P*_heterogeneity_<0.001, *I*^2^ = 70%), TT vs MM (*P*_heterogeneity_<0.001, *I*^2^ = 62%), MT+TT vs MM (*P*_heterogeneity_<0.024, *I*^2^ = 40%), and TT vs MM+MT (*P*_heterogeneity_<0.001, *I*^2^ = 69%) genetic models (Table 5). Also, a publication bias was observed within total population in T vs M (*P*_Egger_ = 0.002), TT vs MM (*P*_Egger_ = 0.058), and TT vs MM+MT (*P*_Egger_ = 0.015) genetic models. Meanwhile, we did not observe any publication bias in Asian population (*P*_Egger_>0.05). Also, the shape of the funnel plot showed no obvious evidence of asymmetry for Asian population (Figure 3). Sensitivity analysis was performed after elimination of one study at a time, and the result was robust (data not shown).

**Table 5:** Results of heterogeneity and publication bias in the meta-analysis*

Group	T vs M			TT vs MM			MT vs MM			MT+TT vs MM			TT vs MM+MT		
	*P*h	*I*^2^	*P*e	*P*h	*I*^2^	*P*e	*P*h	*I*^2^	*P*e	*P*h	*I*^2^	*P*e	*P*h	*I*^2^	*P*e
**Total**	<0.001	70%	0.015	<0.001	62%	0.058	0.519	0%	0.543	0.024	40%	0.123	<0.001	69%	0.002
**Asian**	<0.001	79%	0.087	0.003	65%	0.282	0.544	0%	0.846	0.031	53%	0.476	<0.001	73%	0.094
**Caucasian**	0.055	43%	0.665	0.012	56%	0.423	0.395	5%	0.132	0.387	6%	0.594	0.003	63%	0.123

*Ph – *P*_heterogeneity_ (*P* < 0.1 was considered as a significant difference); *P*e – *P*_Egger_ (*P* < 0.05 was considered as a significant difference).

### In silico results

**Figure 4:**
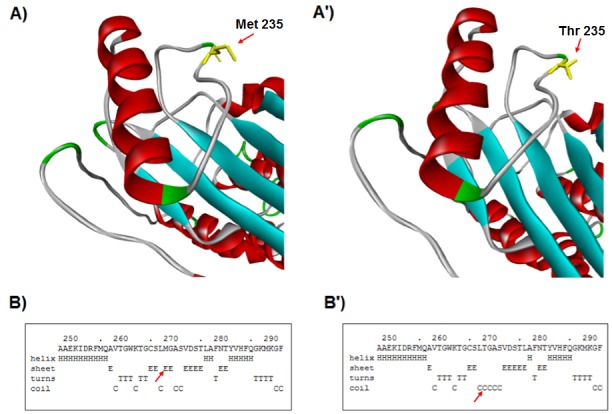
M235T substitution and angiotensinogen (AGT) secondary structure. Representation of Met and Thr residues in position 235 (A&A’). Secondary structure of AGT for 235M and 235T phenotypes in position 268 (B & B’).

**Figure 5:**
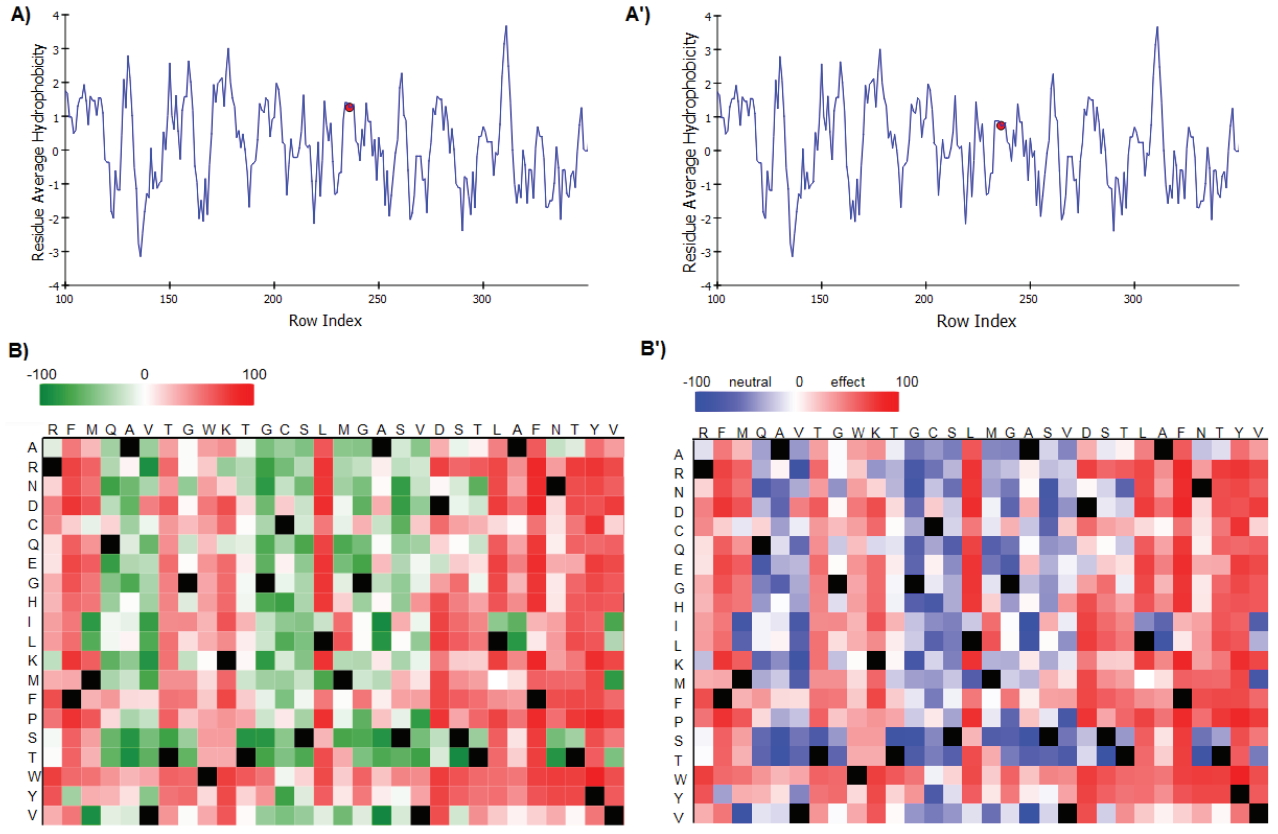
Hydrophobicity, PredictProtein, and SNAP predictions. The hydrophobicity plot for 235M and 235T phenotypes (A&A’). The PredictProtein and SNAP plots for M235T substitution, respectively (B’&B**)**.

The data from Protparam server revealed that the M235T substitution (Figure 4A) changed some physicochemical properties of AGT (Table 6). Grand average of hydropathicity and instability index of the mutant protein were reduced after 235T substitution. Secondary protein structure analysis predicted different secondary structure composition for two AGT variants. Indeed, Thr substitution in position 235, as part of sheet structure of the sequence, was altered to coil structure (Figure 4B). Hydrophobicity analysis revealed that M235T substitution caused a shift in hydrophobicity from 1.26 to 0.74 at residue of 235 (Figure 5A). Polyphen-2 predicted AGT-M235T substitution as a benign mutation in both HumDiv and HumVar models (score, 0.001; sensitivity: 0.99; specificity: 0.15). PredictProtein and SNAP servers revealed a significant effect of M235T substitution on the protein structure (score: 32; expected accuracy: 66%) (Figure 5B).

**Table 6:** Physico-chemical properties for 235M and 235T phenotypes of angiotensinogen protein

Protein phenotype	Molecular weight	Theoretical pI*	Estimated half-life	Instability index	Aliphatic index	GRAVY
235M	53154.2 Da	5.87	30 hours	41.16	99.77	0.065
235T	53124.1 Da	5.87	30 hours	40.99	99.77	0.059

*pI – isoelectric point; grand average of hydropathicity.

## Discussion

This study found TT genotype and T allele to be genetic risk factors for MI. To confirm this result, we also conducted a meta-analysis, which can more accurately determine the impact of a genetic polymorphism on the risk of diseases development and progression (56). The meta-analysis found a statistically significant association between M235T with MI risk in both allelic and recessive models, but it also detected a significant heterogeneity between the studies. Studies in Japanese (13), Chinese (14), Italian (33), and Spanish (35) populations revealed a significant association between the *AGT*-M235T and MI. However, other studies did not observe this association (15-17). This inconsistency might partly be caused by the small sample sizes, especially in the case group, inappropriate for genetic association studies. Another cause may be ethnic and geographic variations. The meta-analysis for Asian populations revealed a significant association between *AGT*-M235T and the MI risk in 2 genetic models, but in 5 genetic models in Caucasian populations there was no significant association.

Different Asian populations also exhibited a great heterogeneity. For example, Chen et al (14), Zhu et al (38), and Ren et al (42), reported a significant association between *AGT*-235T allele and MI risk, whereas Ko et al (16), Frossard et al (28), and Ranjith et al (40) did not observe this association. Such heterogeneity may influence the results of meta-analysis, which is why genetic association studies should be performed within one specific race and ethnicity.

There were 3 meta-analyses that reported erroneous data from the included studies (50-52). Sui and Gao (50) reported no association between *AGT*-M235T and the MI risk, even in the subgroup analysis comprising different ethnic and control sources. However, they (50) included incorrect alleles and genotypes frequencies from 8 studies (29-32,36,39,40,43). Liang et al (51) and Wang and Pan (52) found an association between the polymorphism and MI risk in Asian population only. Liang et al (51), however, incorrectly presented the number of controls from the study of Gardemann et al (29) and the alleles and genotypes frequencies from the study of Hooper et al (37). Wang and Pan (52) incorrectly included two studies (37,39) as Caucasian. In our meta-analysis, all of these mistakes were corrected.

*AGT* gene variants were shown to be associated with hypertension. Also, hypertensive patients with different *AGT* variants showed significantly different plasma concentrations of AGT (57). Thus, *AGT* variant could be a possible candidate for pharmacogenomic renin-angiotensin system blockage intervention (58).

Amino acid substitutions are a common cause of the development of human inherited diseases (59). Therefore, detection of non-synonymous single nucleotide polymorphisms, which lead to amino acid substitutions is essential for understanding the molecular aspects of diseases. A computational analysis of mutations with subsequent alteration in protein function and structure would be beneficial in ranking the disease-causing SNPs with the consequent disorders (60). In this study, we evaluated the effects of M235T substitution on AGT protein by *in silico* tools such as ProtParam, SNAP, PredictProtein, and PolyPhen-2 servers. ProtParam calculates numerous physico-chemical properties inferred from a protein sequence without giving any additional information. PolyPhen2 scoring makes it possible to determine the effects of SNPs on protein phenotypes. It requires input data such as location and variety of amino acid substitution (40). SNAP, as a neural-network based tool, assesses the impact of amino acid changes on proteins. SNAP server uses different biophysical features of the substitution in order to predict the impact of mutation on protein function (38). Although our data from PolyPhen2 recognized M235T substitution as a benign mutation, other *in silico* analyses revealed this substitution as a mutation affecting physicochemical properties, secondary structure, and AGT function.

There are some limitations of our study. In the case-control study, we set the lower age limit for study entry, although cases with earlier disease onset should also be considered. In addition, we did not evaluate factors such as epigenetics, other genes, other mutations, and environmental factors, which may modulate the effects of *AGT*-M235T polymorphism on MI. In the meta-analysis, there was a lack of data from African populations. Second, a lack of original data from the studies included in the meta-analysis restricted further evaluations of the potential interactions that may modulate MI risk, such as gene-environment and gene-gene.

In conclusion, data from the genetic association study revealed that M235T substitution in *AGT* might be a genetic risk factor for MI, especially in Asian population. Also *in silico* analysis revealed that this substitution may affect the AGT structure and function. Future studies in larger populations should assess epigenetics, other genes, other mutations, and environmental factors.
