# Supplementary material for: Angiotensinogen-M235T as a risk factor for myocardial infarction in Asian populations: a genetic association study and a bioinformatics approach
Source: Croat Med J. 2016 Aug;57(4):351–62. doi: 10.3325/cmj.2016.57.351 (PMC5048226; doi:10.3325/cmj.2016.57.351)
Supplement: Supplementary Table 1 [file CroatMedJ_57_s003.pdf]

PRISMA 2009 Checklist

Supplementary Table 1. Checklist of items to include in this meta-analysis

Section/topic	#	Checklist item	Reported on page #
**TITLE**			
Title	1	Identify the report as a systematic review, meta-analysis, or both.	P1; Title page
**ABSTRACT**			
Structured summary	2	Provide a structured summary including, as applicable: background; objectives; data sources; study eligibility criteria, participants, and interventions; study appraisal and synthesis methods; results; limitations; conclusions and implications of key findings; systematic review registration number.	P1; Abstract section
**INTRODUCTION**			
Rationale	3	Describe the rationale for the review in the context of what is already known.	P1-2; The first section of Introduction
Objectives	4	Provide an explicit statement of questions being addressed with reference to participants, interventions, comparisons, outcomes, and study design (PICOS).	P2; The last section of introduction
**METHODS**			
Protocol and registration	5	Indicate if a review protocol exists, if and where it can be accessed (e.g., Web address), and, if available, provide registration information including registration number.	N/A
Eligibility criteria	6	Specify study characteristics (e.g., PICOS, length of follow-up) and report characteristics (e.g., years considered, language, publication status) used as criteria for eligibility, giving rationale.	P4; Meta-analysis paragraph of methods section
Information sources	7	Describe all information sources (e.g., databases with dates of coverage, contact with study authors to identify additional studies) in the search and date last searched.	P4; Meta-analysis paragraph of methods section
Search	8	Present full electronic search strategy for at least one database, including any limits used, such that it could be repeated.	P4; Meta-analysis paragraph of methods section

PRISMA 2009 Checklist

Study selection	9	State the process for selecting studies (i.e., screening, eligibility, included in systematic review, and, if applicable, included in the meta-analysis).	Figure 1
Data collection process	10	Describe method of data extraction from reports (e.g., piloted forms, independently, in duplicate) and any processes for obtaining and confirming data from investigators.	P4; Meta-analysis paragraph of methods section, and Figure 1
Data items	11	List and define all variables for which data were sought (e.g., PICOS, funding sources) and any assumptions and simplifications made.	Table 2
Risk of bias in individual studies	12	Describe methods used for assessing risk of bias of individual studies (including specification of whether this was done at the study or outcome level), and how this information is to be used in any data synthesis.	N/A
Summary measures	13	State the principal summary measures (e.g., risk ratio, difference in means).	P6; The last paragraph of statistical analysis in methods section
Synthesis of results	14	Describe the methods of handling data and combining results of studies, if done, including measures of consistency (e.g., I^2) for each meta-analysis.	P6; The last paragraph of statistical analysis in methods section
Risk of bias across studies	15	Specify any assessment of risk of bias that may affect the cumulative evidence (e.g., publication bias, selective reporting within studies).	P6; The last paragraph of statistical analysis in methods section
Additional analyses	16	Describe methods of additional analyses (e.g., sensitivity or subgroup analyses, meta-regression), if done, indicating which were pre-specified.	P6; The last paragraph of statistical analysis in methods section
**RESULTS**			
Study selection	17	Give numbers of studies screened, assessed for eligibility, and included in the review, with reasons for exclusions at each stage, ideally with a flow diagram.	P7; The first paragraph of meta-analysis in

PRISMA 2009 Checklist

			results section, and Figure 1
Study characteristics	18	For each study, present characteristics for which data were extracted (e.g., study size, PICOS, follow-up period) and provide the citations.	Table 2
Risk of bias within studies	19	Present data on risk of bias of each study and, if available, any outcome level assessment (see item 12).	N/A
Results of individual studies	20	For all outcomes considered (benefits or harms), present, for each study: (a) simple summary data for each intervention group (b) effect estimates and confidence intervals, ideally with a forest plot.	P7-8; The second paragraph of meta-analysis in results section; Figure2
Synthesis of results	21	Present results of each meta-analysis done, including confidence intervals and measures of consistency.	P7-8; The second paragraph of meta-analysis in results section; Table4
Risk of bias across studies	22	Present results of any assessment of risk of bias across studies (see Item 15).	P8; The last paragraph of meta-analysis in results section, and Table5
Additional analysis	23	Give results of additional analyses, if done (e.g., sensitivity or subgroup analyses, meta-regression [see Item 16]).	P7-8; The two last paragraph of meta-analysis in results section, Table4,Table 5, and Figure2
**DISCUSSION**			

Summary of evidence	24	Summarize the main findings including the strength of evidence for each main outcome; consider their relevance to key groups (e.g., healthcare providers, users, and policy makers).	P9; The first paragraphs of discussion section
Limitations	25	Discuss limitations at study and outcome level (e.g., risk of bias), and at review-level (e.g., incomplete retrieval of identified research, reporting bias).	P12; The sixth paragraphs of discussion section
Conclusions	26	Provide a general interpretation of the results in the context of other evidence, and implications for future research.	P12; The last paragraph of discussion section
**FUNDING**			
Funding	27	Describe sources of funding for the systematic review and other support (e.g., supply of data); role of funders for the systematic review.	N/A

From: Moher D, Liberati A, Tetzlaff J, Altman DG, The PRISMA Group (2009). Preferred Reporting Items for Systematic Reviews and Meta-Analyses: The PRISMA Statement. PLoS Med 6(7): e1000097. doi:10.1371/journal.pmed1000097

For more information, visit: **www.prisma-statement.org**.
