# Supplementary material for: Angiotensinogen-M235T as a risk factor for myocardial infarction in Asian populations: a genetic association study and a bioinformatics approach
Source: Croat Med J. 2016 Aug;57(4):351–62. doi: 10.3325/cmj.2016.57.351 (PMC5048226; doi:10.3325/cmj.2016.57.351)
Supplement: Supplementary Table 2 [file CroatMedJ_57_s004.pdf]

SUPPLEMENTARY TABLE 1. Summary OR and 95% CI adjusted for multiple testing using BH-FDR method

Group	T *vs.* M		TT *vs.* MM		MT *vs.* MM		MT+TT *vs.* MM		TT *vs.* MM+MT	
	**OR (95% CI)**	P_{FDR}	**OR (95% CI)**	P_{FDR}	**OR (95% CI)**	P_{FDR}	**OR (95% CI)**	P_{FDR}	**OR (95% CI)**	P_{FDR}
Total	1.12 (1.01-1.25)	0.083	1.17 (0.94-1.46)	0.270	1.03 (0.94-1.13)	0.540	1.06 (0.93-1.22)	0.444	1.24 (1.03-1.50)	0.083
Asian	1.55 (1.10-2.18)	0.030	1.72 (0.95-3.14)	0.127	1.15 (0.86-1.55)	0.352	1.41 (0.88-2.25)	0.190	1.69 (1.13-2.53)	0.030
Caucasian	1.01 (0.92-1.10)	0.941	1.01 (0.81-1.28)	0.941	1.02 (0.92-1.14)	0.941	1.00 (0.90-1.10)	0.941	1.04 (0.83-1.29)	0.941

OR: odds ratio; CI: confidence interval; FDR: *p* value from Benjamini-Hochberg method control for false discovery rate.
